# Function of WAKs in Regulating Cell Wall Development and Responses to Abiotic Stress

**DOI:** 10.3390/plants14030343

**Published:** 2025-01-23

**Authors:** Xiaocui Yao, John Humphries, Kim L. Johnson, Jinhui Chen, Yingxuan Ma

**Affiliations:** 1 State Key Laboratory of Tree Genetics and Breeding, Co-Innovation Center for Sustainable Forestry in Southern China, Key Laboratory of Forest Genetics & Biotechnology of Ministry of Education, International Joint Laboratory on Forest Genetics and Germplasm Innovation, Nanjing Forestry University, Nanjing 210037, China; xhz408@njfu.edu.cn (X.Y.); chenjh@njfu.edu.cn (J.C.); 2 La Trobe Institute for Sustainable Agriculture & Food, Department of Animal, Plant and Soil Science, AgriBio Building, La Trobe University, Bundoora, VIC 3086, Australia; j.humphries@latrobe.edu.au

**Keywords:** wall-associated kinase (WAK), primary cell wall (PCW), secondary cell wall (SCW), cell wall integrity (CWI), pectin, oligogalacturonides

## Abstract

Receptor-like kinases (RLKs) are instrumental in regulating plant cell surface sensing and vascular tissue differentiation. Wall-associated kinases (WAKs) are a unique group of RLKs that have been identified as key cell wall integrity (CWI) sensors. WAK signaling is suggested to be activated during growth in response to cell expansion or when the cell wall is damaged, for example, during pathogen attack. WAKs are proposed to interact with pectins or pectin fragments that are enriched in primary walls. Secondary walls have low levels of pectins, yet recent studies have shown important functions of WAKs during secondary wall development. Several *wak* mutants show defects in secondary wall thickening of the xylem vessels and fibers or the development of vascular bundles. This review will discuss the recent advances in our understanding of WAK functions during plant development and responses to abiotic stresses and the regulation of vascular tissue secondary wall development.

## 1. Introduction

Global climate change presents a challenge for our future society in obtaining sufficient food, fiber, and other important biomaterials from plants [1]. Knowledge of plant growth and stress responses is a fundamental requirement for efficient plant genetic breeding. As the outermost structure of plant cells, the strong but extensible plant cell walls are important for generating intracellular turgor pressure and withstanding mechanical stress [2]. Apart from their key role as physical barriers, it is now well recognized that plant cell walls are dynamic networks that play active roles in regulating cell growth and response to stress. Plant cell wall integrity (CWI) systems are integral to monitoring walls during growth and in response to damage, initiating signaling pathways to maintain structural integrity. The important roles of CWI sensing systems in regulating plant growth and stress tolerance have been identified in various species, including Arabidopsis [3,4], rice [5,6], maize [7], and wheat [8,9]. Key CWI sensors activated upon cell wall damage or wall stress include receptor-like kinases (RLKs), arabinogalactan-proteins (AGPs), GPI-anchored proteins (GPI-APs), and ion channels [10]. A variety of CWI downstream signaling pathways have also been uncovered, such as ethylene signaling, brassinosteroid (BR) signaling, and reactive oxygen species (ROS) signaling pathways [11]. A better understanding of plant cell surface sensing mechanisms can assist novel strategies to develop plants with a higher resilience to external stress.

Wall-associated kinases (WAKs) are members of the RLK superfamily and, since their identification in the 1990s [12], have been recognized as key cell surface sensors during plant growth, abiotic stress, and pathogen invasion. Following the discovery of *WAKs*/*WAKLs* in Arabidopsis, researchers have characterized *WAKs*/*WAKLs* genes in many other plants, including rice (*Oryza sativa*) [5], maize (*Zea mays*) [13], wheat (*Triticum aestivum*) [9,14], barley (*Hordeum vulgare*) [15], tomato (*Solanum lycopersicum* L.) [16,17], potato (*Solanum tuberosum* L.) [18], cotton (*Gossypium* sp.) [19], rose (*Rosa rugosa* Thunb.) [20], tobacco (*Nicotiana tabacum*) [21], and apple (*Malus domestica*) [22]. With the development of genomic and bioinformatical tools, more than 1000 *WAK* genes have been identified from 37 species, ranging from algae to higher plants [23]. Our understanding of WAK functions has been developed through the study of physical interactions with pectin, protein interaction studies, and the investigation of downstream signaling pathways. Much information has been discovered regarding the important roles of WAKs in biotic stress responses, including pathogen infection, as recently reviewed [24,25]. WAKs have been shown to respond to biotrophic and necrotrophic pathogens, bacteria, and insects [14,26,27,28,29]. In recent years, a series of WAK genes involved in defense response have been identified in rice, wheat, and other crops. *Brassica rapa WAK1* (*BrWAK1*) was the first identified and characterized WAK conferring disease resistance to the biotrophic pathogen downy mildew in Chinese cabbage [26]. In wheat, the *TaWAK5* and *TaWAK6* genes are involved in resistance to wheat necrotrophic fungus sharp eyespot disease and leaf rust, respectively [14,27]. In rice, the overexpression of *OsWAK1* can enhance resistance to rice blast fungus [30]. In strawberry, *Fragaria vesca WAK1* (*FvWAK1*), *FvWAK5*, and *FvWAK9* are involved in gray mold resistance caused by the necrotrophic fungus *Botrytis cinerea* [31]. *Glycine max WAK1* (*GmWAK1*) is induced by *Phytophthora sojae* infection and plays a positive regulatory role in soybean response to this pathogen [28]. Thirteen *Beta vulgaris* subsp. *Vulgaris* L. *WAK/WAKL* (*BvWAK/WAKL*) genes were found to be upregulated exclusively in the resistant cultivar, which suggests a potential involvement of these genes in the resistance of sugar beet to the beet cyst nematode (*Heterodera schachtii Schmidt*) [29].

In comparison, the role of WAKs during plant growth and development is far less understood. For example, the novel roles of WAKs in regulating vascular tissue secondary wall development have only recently been uncovered [32]. New molecular mechanisms, such as the regulation of ethylene and brassinosteroid (BR) signaling pathways by WAKs, have also been suggested [33,34]. Here, we aim to summarize the recent progress in the research of WAKs with an emphasis on wall development and response to abiotic stresses.

## 2. WAK Domain Features

WAKs possess the characteristics of RLKs, such as having an extracellular ligand-binding domain, a transmembrane helix, and an intracellular kinase domain. WAK/WAK-like (WAKL) protein sequences are characterized by having a Galacturonan-Binding domain (GUB domain), an Epidermal Growth Factor domain (EGF domain), a transmembrane domain, and cytoplasmic protein kinase [12]. The EGF domain is implicated in protein–protein interactions [35]. EGF domains can be classified into those that bind calcium (EGF-Ca) and those that do not (EGF2). These distinctions are based on their consensus sequence [36]. In certain instances, the EGF domain is necessary for the regulation of ligand binding [35]. The non-EGF portion of the extracellular domain in the WAK protein typically encompasses a conserved domain, designated the GUB domain, which has been demonstrated to covalently bind to de-esterified homogalacturonan (the primary component of cell wall pectin) [37]. WAKLs display sequence similarity to WAKs and contain GUB and kinase domains, yet are more variable in the combination of other domains and may lack EGF domains and transmembrane regions [38]. There have been inconsistencies in the naming conventions of WAKs and WAKLs in the literature, and a recent review comprehensively addresses this issue [39].

The WAK gene family has been found in a variety of angiosperms, such as dicots Arabidopsis (27 WAKs/WAKLs) [12], cotton (128 WAKs/WAKLs) [40], rose (69 WAKs/WAKLs) [20], tomato (29 WAKs/WAKLs) [16], monocots rice (125 WAKs/WAKLs) [41], *Brachypodium distachyon* (115 WAKs/WAKLs) [42], barley (91 WAKs/WAKLs) [15], basal angiosperms *N. colorata* (11 WAKs/WAKLs), gymnosperms *P. abies* (7 WAKs/WAKLs), *G. montanum* (4 WAKs/WAKLs), pteridophytes *A. filiculoides* (5 WAKs/WAKLs), *S. cucullata* (5 WAKs/WAKLs), lycophytes *S. moellendorffii* (1 WAK/WAKL), and bryophyte *P. patens* (2 WAKs/WAKLs) [23]. It is possible that WAK family genes have undergone lineage-specific amplification in angiosperms [23]. During the process of speciation, there was a divergence in sequencing, with monocots and dicots diverging into different branches. Rice and Arabidopsis are important model plants for monocot and dicot crops, respectively. The expansion of Arabidopsis WAK/WAKLs is due to both local tandem duplications and large-scale genomic duplications [38]. In comparison to the 27 Arabidopsis WAKs/WAKLs, the rice genome exhibits a nearly 5-fold increase in the number of OsWAKs [41]. This expansion does not appear to be attributable to the larger genome size of rice in isolation. Considering that monocot cell walls typically contain less pectin compared to dicots, the increase in WAK numbers in monocots is intriguing. WAKs/WAKLs in monocots potentially reflects different requirements for disease and abiotic stress tolerance pathways and could be required to fine tune responses to changes in pectin composition or other stress-induced cell wall proteins/carbohydrates, for example, by acting in complexes [43]. Detailed information about WAK/WAKL bioinformatics has been reviewed in a recent review [39] and will not be discussed further here.

The first WAK family to be characterized was from Arabidopsis, and phylogenetic studies grouped the five Arabidopsis AtWAKs together and the AtWAKLs in a separate clade, suggesting distinct evolutionary patterns [23] (Supplementary Figure S1). Furthermore, previous studies have classified WAKs/WAKLs into four groups on the basis of structural domains, and five WAK (WAK1-WAK5) genes were classified into group I, distinguished from WAKLs [23]. A comparison of the amino acid sequences of WAK and WAKL reveals a substantial discrepancy between them, primarily in the WAK-GUB domain (WAK-GUB), EGF2-like region (EGF-2), transmembrane domain (TM), and cytoplasmic protein kinase (protein kinases) (Supplementary Figure S2). The phylogenetic and structural domain differences may lead to functional specialization. *AtWAK* expression has been identified in vegetative tissues during developmental stages with expression levels higher than those of *AtWAKLs* [23]. Furthermore, biotic stresses, including pathogen infection and damage, have been observed to induce *AtWAKs*. Transgenic plants overexpressing *AtWAK1* have shown elevated resistance to the necrotrophic pathogen *Botrytis cinerea* [44]. In comparison, the expression levels of *AtWAKLs* under salt and drought stress were higher than those of *AtWAKs* [23], suggesting that WAKLs may be more specialized for abiotic stress responses and WAKs for growth and defense.

## 3. WAKs Regulating Primary Wall Expansion

The WAK/WAKL family has been shown to play important roles in regulating plant growth and development in a variety of plant species by associating with primary cell walls. The fundamental structure of primary cell walls (PCWs) is composed of cellulose microfibrils embedded within a matrix of hemicelluloses, primarily xyloglucans in dicots, pectins, and (glyco)proteins [45]. The commelinid monocots, such as grasses, exhibit a comparable PCW structural organization to dicots, albeit with a distinctive matrix comprising glucuronoarabinoxylans and (1,3;1,4)-β-D-glucans [45]. Rice and Arabidopsis are important model plants for monocot and dicot crops, respectively, and will be used as initial examples to demonstrate WAK/WAKL functions in this review.

WAKs are known to be able to bind to pectin in cell walls [46,47]. Studies in Arabidopsis suggest that WAKs can bind homogalacturonan pectin and oligogalacturonide (OG) pectin fragments [48]. AtWAK1 and AtWAK2 have a preference for short OG with a degree of polymerization of 9–15 but also bind to longer pectin polymers in a calcium-dependent manner with lower affinity [37,49]. In Arabidopsis, *AtWAK1* and *AtWAK2* are the most abundantly expressed *WAKs* and are predominantly located in leaves, roots, flowers, and stems [46]. It was demonstrated that cell expansion is influenced by *AtWAK1* and *AtWAK2* though gene knockout/knockdown studies [47,50]. The silencing of *AtWAK2* resulted in a reduction in the size of the rosette leaves of the plant, and no discernible phenotypes were observed in *WAK1*-specific antisense plants [46]. Invertase was suggested as a downstream target of WAK2 signaling pathways during cell expansion via regulating cell turgor pressure [46]. An analysis of *ESMERALDA1* (*esmd1*) mutants, defective in a putative O-fucosyltransferase predicted to modify EGF domains, suggest that O-fucosylation plays an important function in regulating WAK2 signaling [51]. *AtWAK4* was shown to be expressed in all organs at lower levels than *AtWAK1* and *AtWAK2* [52], and antisense *AtWAK4* resulted in a reduction in WAK protein levels and the inhibition of cell elongation. A range of pleiotropic effects were observed in antisense *AtWAK4*, including the development of shorter primary roots, smaller rosette leaves, compressed inflorescence stems, unopened micro-flowers, and shorter appendages [52]. The molecular mechanisms underlying the action of WAK4 require further investigation.

OG triggers a defense response that accumulates ROS through the activation of NADPH oxidase AtRbohD and MAPK-mediated activation of defense gene expression [53]. The direct interaction between AtWAK1 and OGs and the pivotal role of AtWAK1 in plant immunity were initially elucidated in an experiment involving transgenic Arabidopsis plants expressing a chimeric-receptor-like protein comprising an ectodomain of AtWAK1 and a cytoplasmic kinase domain of EF-Tu receptor (EFR) [44]. However, there is a paucity of clear genetic evidence on the role of WAKs in OG responses. A recent report using clustered, regularly interspaced short palindromic repeats (CRISPR) to target all five *AtWAKs* suggests that their role in OG perception is complex [54]. In combination with immunoassays for early and late pattern-triggered immunity, it was found that WAK is unnecessary for OG-induced signal transduction and the initiation of an immune response, suggesting that WAK is not a bona fide OG receptor. A WAK locus deletion experiment showed that OG, bacterial flagellin, and chitin-induced ROS burst were reduced, suggesting they may act in parallel pathways [51]. It is possible that WAKs fine tune the activity of an oxidative burst protein or alter the cell wall such that the true receptors can be activated. Genetic evidence from other species also showed that WAKL functions during pathogen resistance, suggesting potential coordination of WAKs and WAKLs signaling pathways during defense. The mechanism by which WAK promotes immunity and its true ligands remains to be determined.

**Figure 1:**
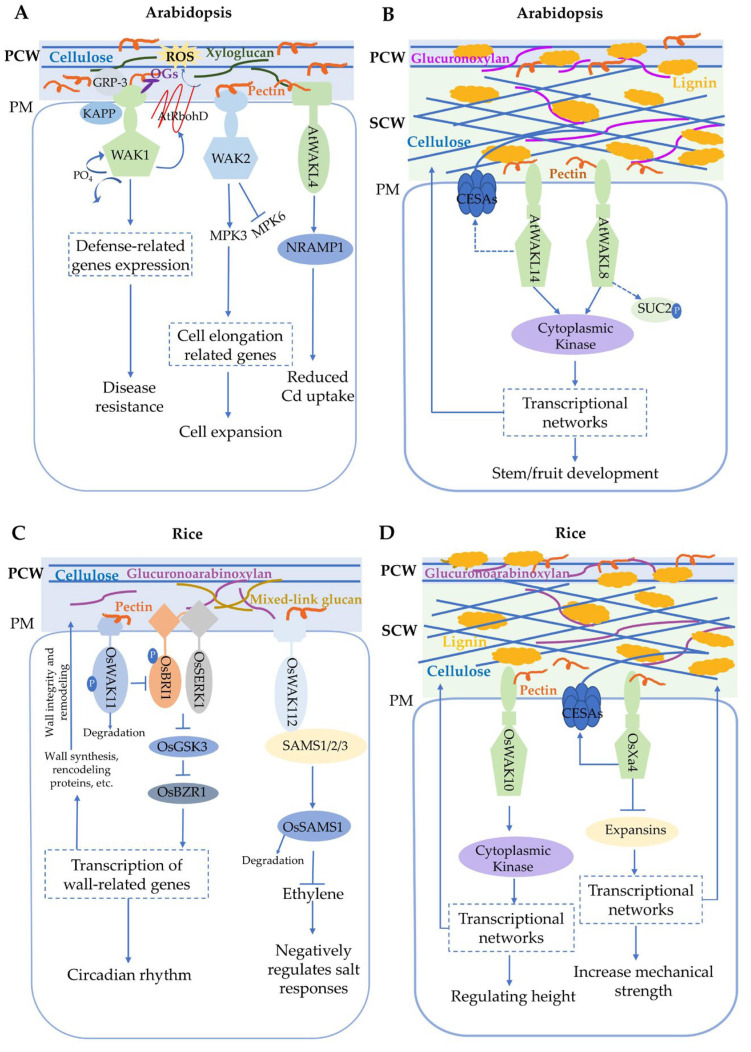
A model for Arabidopsis and rice WAKs regulating primary and secondary wall signaling in plants. (**A**) In Arabidopsis, AtWAK1, Glycine-Rich Protein 3 (AtGRP-3), and Kinase-Associated Protein Phosphatase (KAPP) are associated with a multimeric complex of 500 kDa. WAK1 senses pectin fragments of oligogalacturonide (OG) and triggers a defense response that accumulates ROS through activation of NADPH oxidase AtRbohD and MAPK-mediated activation of defense gene expression. Pectin binding activates WAK kinase and the subsequent activation of Mitogen-activated Protein Kinase 3 (MPK3), leading to the regulation of genes involved in cell expansion. MPK6 is either repressed or not activated. AtWAKL4 interacts with and phosphorylates the Cd transporter protein Natural-Resistance-Associated Macrophage Protein 1 (NRAMP1), resulting in enhanced degradation of NRAMP1, which ultimately reduces Cd uptake. (**B**) AtWAKL14 and AtWAKL8 may interact with cell wall pectin, activating cytoplasmic kinases and downstream signaling pathways to regulate vascular tissue differentiation and SCW development via transcriptional networks, influencing stem and fruit development. (**C**) In rice, OsWAK11 was found to be capable of binding directly to the BR receptor brassinosteroid insensitive 1 (OsBRI1) and phosphorylate the receptor. OsWAK11 binding is proposed to compete with and prevent binding of the co-receptor Somatic Embryogenesis Receptor-like Kinase 1 (OsSERK1). Inhibition of the kinase Glycogen Synthase Kinase 3 (OsGSK3) allows the accumulation of the transcription factors Brassinazole Resistant 1 (OsBZR1) in a dephosphorylated active form and inhibits its activity. OsWAK11 demonstrated stability under light conditions but underwent degradation under dark conditions, thereby releasing the inhibition of OsBARI activity. OsWAK112 negatively regulates plant salt responses by inhibiting ethylene production, possibly via direct binding with S-adenosyl-L-methionine synthetase (SAMS) 1/2/3 (SAMS1/2/3). (**D**) OsWAK10 and OsXa4 interact with cell wall pectin, activating cytoplasmic kinases and downstream signaling pathways to regulate vascular tissue differentiation and SCW development via transcriptional networks.

Although the binding of AtWAKs to OG is unclear during defense, pectin binding appears to be involved in regulating cell expansion and stress response. These different responses likely occur via different WAK protein complexes. AtWAK1, AtGRP-3, and Kinase-Associated Protein Phosphatase (KAPP) are associated with a multimeric complex of 500 kDa [55]. KAPP is a regulator that is involved in the receptor-like kinase (RLK) signaling pathway and has been shown to interact with a number of plant RLKs. This interaction is thought to be a key step in signal perception and transduction. MPKs form a major signaling link between cell surface receptors and both transcriptional and post-transcriptional regulation in eukaryotes [56]. Protoplasts from plants homozygous for the null allele *wak2-1* showed a reduction in the activation of MPK3 [57]. Pectin can activate the genes required for MPK3 (but not MPK6) and cell elongation in a WAK2-dependent manner [47] (Figure 1A). In the presence of OG, WAKs may alter their signaling path to help effect the stress response by also activating MPK6. WAK binding of pectin likely activates cell expansion pathways, and OGs are proposed to interfere with pectin binding and initiate different pathways.

In rice, silencing of the *OsiWAK1* gene resulted in dwarf plants as a cause of reduced size of leaves, flag leaves, internodes, and panicles [58]. The development of root primordia during germination, root hairs, and lateral rooting was also affected. *OsWAK112* is expressed in various tissues throughout the plant, including the coleoptiles, ligules, leaf blades, stems, roots, and the radicles of germinated seeds, indicating a broad role in plant development and stress response [33]. *Grain Weight and Number 1* (*GWN1*) encoded the OsWAK74 protein kinase in rice, with high expression being observed in inflorescences. *GWN1* negatively regulated grain length and weight by influencing cell proliferation in the spikelet hulls. Additionally, *GWN1* negatively affected grain number by influencing secondary branch number and finally increased plant grain yield [59]. OsDEES1, a WAK protein in rice, was shown to play a role in the development of the female gametophyte [60]. Silencing of OsDEES1 resulted in a high percentage of female sterility. Nevertheless, no studies have shown the underlying molecular mechanisms behind the observed increase in sterility. In a study of a regulator of rice cell elongation, OsWAK11 was revealed to have a role in modulating BR signaling in response to changes in the methyl esterification of pectin in the rice cell wall [61]. OsWAK11 was found to be capable of binding directly to the BR receptor brassinosteroid insensitive 1 (OsBRI1) and was also able to phosphorylate the receptor at a specific site (residue Thr752) (Figure 1C). This resulted in the receptor being unable to interact with co-receptor Somatic Embryogenesis Receptor-Like Kinase 1 (OsSERK1). The kinase Glycogen Synthase Kinase 3 (OsGSK3) was subsequently inhibited in its signaling function [61]. Inactivation of OsGSK3 allows the accumulation of the transcription factor Brassinazole Resistant 1 (BZR1) in a dephosphorylated active form and inhibits its activity. Furthermore, OsWAK11 demonstrated stability under light conditions but underwent degradation under dark conditions, a process influenced by alterations in the ratio of methyl-esterified pectin to dimethyl-esterified pectin, which resulted in fluctuations in BR signaling in plants during the diurnal cycle (Figure 1C). This indicates that OsWAK11 serves as a cell wall monitor that regulates cell elongation and adaptation to environmental changes.

An alternative downstream signaling pathway has been suggested recently in wheat. A single Ala to Val amino acid substitution reduces the protein stability of TaWAK2, leading to a narrow-leaf phenotype [62]. TaWAK2 directly interacts with, and phosphorylates, Narrow Leaf 1 (TaNAL1), a trypsin-like Ser/Cys protease. Phosphorylated TaNAL1 is then involved in the degradation of the zinc finger transcription factor Drought and Salt Tolerance (TaDST), which acts as a repressor of leaf expansion by activating the expression of the cytokinin oxidase gene Cytokinin oxidase/dehydrogenase 9 (TaCKX9) and triggering cytokinin degradation in vivo. This indicates that TaWAK2-TaNAL1-TaDST signaling pathway regulates leaf width via cytokinin signaling in wheat.

The identification of novel WAK signaling pathways, including ROS, BR, and ethylene, have brought us more insights into WAKs’ functional mechanisms; however, it has also provoked the question of whether a single WAK can trigger different downstream signaling pathways under different circumstances or if there are distinct downstream signaling pathways for different WAKs. In addition, it remains to be explored whether (and how) pectins and OGs regulate cell expansion via competing for binding with the same WAKs/WAKLs or through different WAK receptors and signaling pathways.

## 4. WAKs Regulating Secondary Wall Development

Upon cessation of cell expansion, some cell types, such as vascular tissues, undergo differentiation, and their walls are thickened through the deposition of cellulose and hemicelluloses. In dicots, secondary wall hemicelluloses include glucuronoxylans and heteromannans, and, in commelinid monocots, glucuronoarabinoxylans and heteromannans. Lignin can also be a major component of secondary cell walls (SCWs), providing rigidity, waterproofing, and strength [45]. It has been thought that SCWs have no CWI mechanism, or at least no WAK/WAKL-related CWI pathways, considering that pectin typically represents a minor component of SCWs [63]. However, GUS (β-glucosidase) staining showed that some WAKs/WAKLs from Arabidopsis, rice, maize, and tomato were highly expressed in vascular tissues during both development and under stress [32,49,64]. RNA-seq or qRT-PCR analysis also showed that some WAKs are highly expressed in woody stems [65]. In recent years, mutant analysis in Arabidopsis, rice, and maize have provided substantial evidence of WAKs/WAKLs regulating SCW development [7,32,65,66].

**Figure 2:**
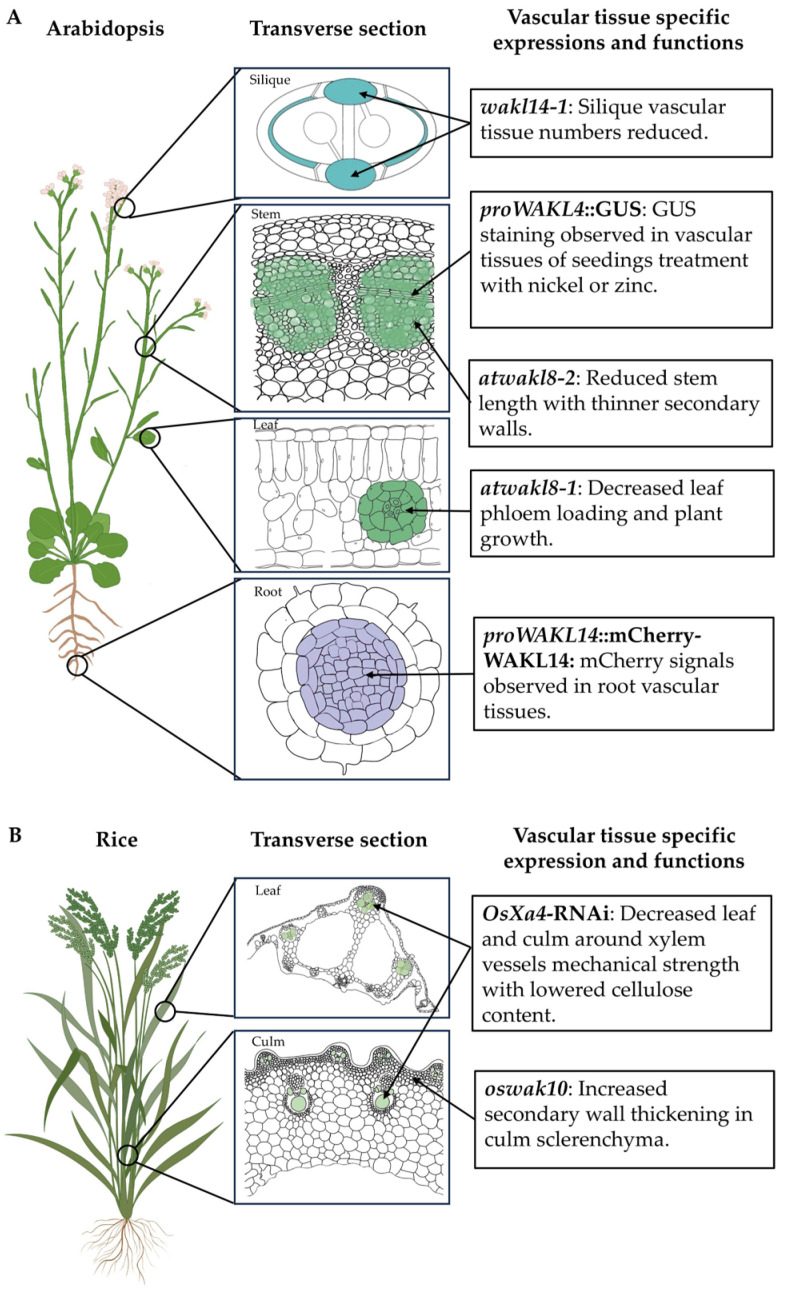
Summary of expression patterns and biological functions of WAKs involved in secondary wall development in Arabidopsis and rice. Expression profiles of *WAKs* in shoot meristem, flower, silique, stem, leaf, and root, and transgenic plant phenotypes in Arabidopsis (**A**) and rice (**B**). Colored cells indicate tissue expression patterns of WAKs in vascular tissues.

*AtWAKL14* is specifically expressed in vascular tissues and regulates vascular tissue development (Figure 2A) [32]. In *atwakl14-1* mutants, the number of xylem vessels (XVs) and Interfascicular Fiber (IF) cells are reduced; however, SCW thickness and composition remain largely unaltered. Similarly, *SlWAKL2*-RNAi (*AtWAKL14* orthologous gene in tomato) transgenic plants showed reduced XV and IF cell numbers in their fruit [32]. *AtWAKL14* could play a role in regulating the differentiation of cells with SCWs, such as those of the vasculature. It is possible that cell-wall-sensing proteins and pathways may differ from those of the primary wall during the process of SCW development. Previous studies showed that AtWAKL14 and SlWAKL2 may interact with pectin and OGs to activate cytoplasmic kinases and trigger downstream signaling pathways, thereby regulating vascular tissue differentiation, which in turn determines the number of XVs and IFs and governs the development of the SCW through transcriptional networks [32] (Figure 1B). Further studies are required to elucidate the specific downstream signaling pathways and molecular mechanisms involved.

AtWAKL8 has also been demonstrated to play a significant role in regulating SCW development in Arabidopsis stems (Figure 2A) [65]. The *atwakl8-2* mutant displayed a reduction in the thickness of the xylem and fiber cell walls. *AtWAKL8* was identified to be expressed in leaf vascular tissues [67]. WAKL8 is proposed to interact with and phosphorylate sucrose transporter 2 (SUC2) and positively regulate phloem loading [67]. Further experimentation is required to ascertain whether WAKL8 phosphorylates SUC2 (and other sucrose transporters) in stem tissues, potentially regulating the supply of sucrose necessary for stem SCW development (Figure 1B). Furthermore, the proteins that interact with WAKL8 and the downstream signaling pathways must be identified [65]. *WAKL8* also undergoes alternative splicing, and a similar alternative splicing of WAKs was reported in maize *ZmWAK-RLK1* [7]. ZmWAK-RLK1, a protein commonly found in plant innate immune receptors, plays an important role in resistance to adaptive pathogens [7]. Despite the suggestion that both ZmWAK-RLK1 and AtWAKL8 truncated versions are nonfunctional, it is not possible to rule out the possibility that these spliced transcript versions play a role when plants are exposed to specific growth and/or stress conditions. As these truncated proteins still contain extracellular domains, they may compete with pectins and pectin fragments for binding sites, thereby reducing the signaling strength under certain circumstances [65]. It remains to be determined whether the alternative splicing of WAKs/WAKLs is conserved in different species and what mechanisms are involved in regulating the splicing of *WAK* transcripts.

In rice, OsWAK10 might be involved in regulating signaling pathways in which two WAK10 ectodomain variants bind pectic oligosaccharides with different affinities [66] (Figure 1D and Figure 2B). The binding of WAKL10 with pectic oligosaccharide regulates its phosphorylation activity and affects secondary wall deposition and stem height. Phylogenetic analyses have demonstrated that the majority of OsWAKs and AtWAK/WAKLs genes are distributed in species-specific branches and evolve in a species-specific manner [41]. It is noteworthy that OsWAK10 and AtWAKL14 are situated in the same evolutionary branch, indicating the potential for shared ancestral genes [41]. This suggests that WAK genes situated within the same evolutionary branch may exhibit analogous functions, offering a novel avenue for the identification of WAK genes that regulate secondary wall development.

OsXa4, encoding a cell-wall-associated kinase, was shown to be predominantly located in the parenchymal cell wall encircling xylem vessels in rice leaf tissue (Figure 2B) [68]. The expression of Cellulose Synthase (CESA) family genes, *CESA4*, *CESA7*, and *CESA9*, responsible for secondary wall synthesis in rice, was elevated in Xa4-carrying transgenic plants relative to the wild type. In contrast, the expression levels of the two α-expansin genes, *EXPA1* and *EXPA5*, were lower than those of the wild type in Xa4-carrying transgenic plants. This suggests that Xa4 has the potential to enhance mechanical strength and, thus, reinforce the cell wall by promoting cellulose synthesis and inhibiting expansin expression (Figure 1D) [68].

Studies of WAK/WAKL in Arabidopsis, rice, maize, and tomato have provided substantial evidence of their key functions during SCW development. However, our current understanding of WAK/WAKL interacting partners and downstream signaling pathways is still underdeveloped compared to that of those in primary walls. ROS signaling has been shown as key a WAK/WAKL downstream pathway in primary walls and also during defense against pathogen invasions [24]. ROS is important for lignin polymerization in SCWs and could be a mechanism by which WAK/WAKLs influence secondary wall development and mechanical properties. In addition, MPK has been shown to regulate the phosphorylation state and activity of SCW transcription factors during poplar wood development [69], suggesting a possibility that WAK/WAKL may regulate SCW development via MPK cascades, similar to primary walls. Carbon availability is important for cell wall polysaccharides synthesis. As AtWAKL8 can regulate sucrose transportation via SUC2 [65], and AtWAK2 can regulate cell expansion via invertase [47], it is possible that WAK/WAKL may regulate SCW development via sugar metabolism. Moreover, it is well recognized the BR signaling pathways are important for plant vascular tissue development [34]. The evidence that OsWAK11 can regulate BR signaling pathways provides an alternative possibility of how WAKs/WAKLs regulate vascular tissue differentiation and SCW development. Further studies into the specific downstream pathways and molecular mechanisms of WAKs in the SCW signaling pathway could contribute to the improvement of the WAK signal transduction network and provide novel strategies for the genetic breeding of plants.

## 5. WAKs Regulating Abiotic Stress Responses

Plants are susceptible to stress caused by environmental changes during their growth and development [70]. The cell wall represents the initial barrier to the perception of external stimuli. WAKs/WAKLs have been recognized as sensors of the extracellular environment and triggers of intracellular signaling [16]. Previous studies have demonstrated that various WAKs/WAKLs are involved in the response to abiotic stress, including salt stress and metal stress (Table 1).

WAKs/WAKLs play important but varying roles in response to salt stress. For instance, the *atwakl10* mutant plants showed reduced germination percentages under high salinity conditions compared to wild-type plants [4]. Additionally, the *SlWAK1* gene plays an important role in response to salt stress, particularly in maintaining osmotic balance and ion homeostasis [71]. The disruption of SlWAK1 leads to increased salt sensitivity, likely due to compromised osmotic adjustment and ion regulation, although the exact mechanisms and pathways involved require further investigation [71]. *GhWAKL26* has been shown to play a regulatory role in cotton salt tolerance, modulating the homeostasis of Na^+^ and K^+^ [72]. A similar function has been ascribed to *GbWAK5* in enhancing salt stress resistance in *G. barbadense* cotton, again by modulating ion homeostasis [73]. In *Nitraria sphaerocarpa*, the expression of *WAK4* was increased after a long exposure to salt [74]. Rice OsWAK112 has a broad role in plant development and stress response and is expressed in various tissues throughout the plant. The overexpression of *OsWAK112* leads to increased sensitivity to salt stress, as evidenced by reduced shoot lengths and fresh shoot weights under salt treatment conditions [33]. OsWAK112 is an active kinase that interacts with S-adenosyl-L-methionine synthetase (SAMS) 1/2/3 (SAMS1/2/3), promoting OsSAMS1 degradation and reducing ethylene production under saline conditions (Figure 1C). These examples demonstrate the critical role of WAK in the salt stress response of plants and help to elucidate the complex mechanisms of plant adaptation to salt stress. Despite these studies, the molecular mechanisms by which WAKs function in response to salt stress remain to be further investigated. For example, OsWAK112 negatively regulates salt tolerance, whereas SlWAK1 and AtWAKL10 positively regulate salt tolerance, suggesting that WAKs have distinct functions in regulating the pathways of salt stress depending on the wall context and signaling pathways.

WAKs/WAKLs play important roles in the response to heavy metals. For example, mRNA and protein levels of AtWAK1 were found to increase rapidly in response to aluminum treatment, and the overexpression of AtWAK1 delayed aluminum-stress-induced root growth inhibition [75]. Similarly, AtWAKL4 is involved in the heavy metal response of Arabidopsis, and its expression is associated with plant tolerance to nickel (Ni^2^⁺) and zinc (Zn^2^⁺) [76]. As analyzed using GUS staining, *AtWAKL4* was mainly expressed in lateral root initiation sites and root vascular tissues, and copper (Cu^2^⁺) and nickel (Ni^2^⁺) treatments significantly enhanced WAKL4::GUS activity. Recent studies show that WAKL4 also mediates a plant-activated reduction in cadmium (Cd) accumulation [77]. AtWAKL4 interacts with and phosphorylates the Cd transporter protein Natural-Resistance-Associated Macrophage Protein 1 (NRAMP1) at the Tyr488 site, resulting in enhanced ubiquitination and vesicle-dependent degradation of NRAMP1, which ultimately reduces Cd uptake. The AtWAKL4-NRAMP1 module enables plants to respond positively to Cd and limit its uptake, providing information for future molecular breeding of low Cd-accumulating crops or vegetables (Figure 1A). The molecular mechanism of WAKs in response to salt and heavy metals stress may facilitate an understanding of the adaptation mechanisms of plants under adverse conditions. This provides a theoretical basis for improving plant tolerance to adverse environments, such as salinized and heavy-metal-contaminated soils.

OsWAK11 plays a crucial role in the response to copper (Cu) toxicity in rice [78]. The expression of *OsWAK11* is downregulated in response to excess Cu, and it is involved in Cu detoxification. *OsWAK11-RNAi* plants exhibit a higher level of wall methyl esterification, which may account for the diminished accumulation of Cu in the cell wall in OsWAK11 plants. The regulatory pathway of OsWAK11 likely involves its interaction with the cell wall pectin methyl esterification, influencing the binding capacity of the cell wall for Cu. Further investigation of the downstream targets of OsWAK11 and the elucidation of the signaling pathways will be essential to understanding the molecular mechanisms.

In *Pisum sativum*, the expression pattern of *WAK* genes in response to B deficiency was similar to that of Al toxicity, with most *PsWAKs* being upregulated [64]. *PsWAK5*, *PsWAK9*, and *PsWAK14* were more specific for both B-deficiency and Al toxicity, suggesting that *PsWAKs* may play a key role in the perception of cell wall integrity under Al toxicity or B-deficiency, as well as in the regulation of Al tolerance in peas [64].

Additionally, WAKs/WAKLs also play a role in cold and heat stresses (Table 1). Silencing *CaWAKL20* improved the heat tolerance of pepper [79]. WAKs could bind either oligogalacturonides or pectin in the cell wall in response to cold stress, indicating a potential role of the cell wall as a cold stress sensor [80]. In cotton, the expressions of *GhWAK9*, *GhWAK12*, *GhWAK14*, *GhWAKL46*, and *GhWAKL47* were significantly upregulated in response to cold, heat, salt, and drought stresses. *GhWAKL17* expression was upregulated in the initial period after cold treatment [19]. In *Cannabis sativa* L., *CsWAK12* acts as a negative regulator, reducing the cold tolerance of transgenic Arabidopsis by mediating the CBF pathway [81]. *OsWAK112d* enhanced rice resistance to cold stress [80]. The response of WAKs to abiotic stress, especially salt and metal stresses, provides insight into how plants perceive and respond to external abiotic stresses and offers a theoretical foundation for enhancing plant resistance. As receptor kinases that sense and transmit environmental signals, WAK/WAKLs play pivotal roles in the signaling pathways of plants in response to salt stress. Although some proteins that interact with WAKs have been identified, there are likely other crucial interacting proteins that have yet to be identified. Consequently, a more profound investigation of the intricacies of protein–protein interactions of the WAKs signaling pathway and their subsequent signaling mechanisms would offer a more comprehensive theoretical foundation for plant growth regulation and breeding.

**Table 1:** Functions of WAKs/WAKLs under different abiotic stress responses across various plant species.

Genes	Species	Types	Functions	References
Salt stress				
*AtWAKL10*	*Arabidopsis thaliana* L.	WAKL	Positively regulated the salt tolerance of Arabidopsis	[4]
*GhWAKL26*	*Gossypium hirsutum*	WAKL	Enhanced plant resistance to salt stress in cotton by regulating the balance of Na^+^ and K^+^ ions	[72]
*GhWAK9* *GhWAK12*	*Gossypium hirsutum*	WAK	Significantly upregulated in response to salt stress	[19]
*GhWAKL46* *GhWAKL47*	*Gossypium hirsutum*	WAKL		
*GbWAK5*	*Gossypium barbadense*	WAK	Regulated the balance of Na^+^ and K^+^ ions by affecting the expression of ion transport genes, thereby enhancing the salt tolerance of cotton seedlings	[73]
*OsWAK112*	*Oryza sativa*	WAK	Negatively regulated the salt tolerance of rice	[33]
*SlWAK1*	*Solanum lycopersicum* L.	WAK	Controls osmotic and metabolic homeostasis in tomato under high salt stress, negatively regulating salt sensitivity	[71]
*NsWAK4*	*Nitraria sphaerocarpa*	WAK	Significant increase in expression levels following long exposure to salt	[74]
*BdWAK2* *BdWAK10*	*Brachypodium distachyon*	WAK	Significant increase in expression levels in response to sodium salicylate and salt treatments	[42]
*BdWAK72*	*Brachypodium distachyon*	WAK	Sensitivity to salt stress	[42]
Heavy metal stress				
*AtWAK1*	*Arabidopsis thaliana* L.	WAK	Overexpression lines restored root growth inhibition by Al stress	[75]
*AtWAKL4*	*Arabidopsis thaliana* L.	WAKL	Vital role in root mineral nutrient responses, such as Na^+^, K^+^, Cu^2+^, and Zn^2+^	[76]
*OsWAK124*	*Oryza sativa*	WAK	Functions in (heavy) metal stress responses, such as Cd^2+^, Cu^2+^, and Al^3+^	[82]
*OsWAK11*	*Oryza sativa*	WAK	Regulated plant response to heavy metal stress and wounding	[6]
*PsWAK5* *PsWAK9* *PsWAK14*	*Pisum sativum*	WAK	Significant increase in expression levels in Al-sensitive cultivar Hyogo	[64]
Cold stress				
*OsWAK112d*	*Oryza sativa*	WAK	Enhanced rice resistance to cold stress	[80]
*CsWAK12*	*Camellia sinensis*	WAK	Negatively regulated cold tolerance	[81]
*GhWAK9* *GhWAK12*	*Gossypium hirsutum*	WAK	Significantly upregulated in response to cold stress	[19]
*GhWAKL46* *GhWAKL47*	*Gossypium hirsutum*	WAKL		
*GhWAKL17*	*Gossypium hirsutum*	WAKL	Expression upregulated at the beginning of cold treatment	[19]
Heat stress				
*CaWAKL20*	*Capsicum annuum* L.	WAKL	Negatively regulated thermotolerance in pepper	[79]
*GhWAK9* *GhWAK12*	*Gossypium hirsutum*	WAK	Significantly upregulated in response to heat stress	[19]
*GhWAKL46* *GhWAKL47*	*Gossypium hirsutum*	WAKL		
Drought stress				
*GhWAK9* *GhWAK12*	*Gossypium hirsutum*	WAK	Significantly upregulated in response to drought stress	[19]
*GhWAKL46* *GhWAKL47*	*Gossypium hirsutum*	WAKL		

## 6. Materials and Methods

### 6.1. Strategy for the Literature Search

The information reported in the current paper was collected from a literature search using various computerized databases such as PubMed and Web of Science. Keywords such as wall-associated kinases (WAK); primary cell wall (PCW); secondary cell wall (SCW); cell wall integrity (CWI); pectin; and oligogalacturonides were used interchangeably.

### 6.2. Data Analysis

The amino acid sequences of AtWAKs and AtWAKLs of *Arabidopsis thaliana* were downloaded from the Arabidopsis Information Resource (TAIR—Home (https://www.arabidopsis.org/)), accessed on 18 January 2025. The evolutionary tree was generated using MEGA11 with the neighbor-joining method (1000 bootstrap replicates). Multiple comparison analysis of AtWAKs and AtWAKLs were conducted with DNAMAN. Based on previous studies of WAK proteins in Arabidopsis, WAK proteins contain four typical conserved domains, including a signal peptide, an EGF domain, a transmembrane domain, and a protein kinase domain; therefore, CD Search (Conserved Domain Search Service (https://www.ncbi.nlm.nih.gov/Structure/cdd/wrpsb.cgi), accessed on 18 January 2025) results were confirmed by the identification of the four typical domains.

## 7. Conclusions

Analyzing the role of WAKs/WAKLs during cell expansion will provide a foundation for a comprehensive understanding of the molecular mechanisms underlying plant cell expansion and its impacts on overall plant growth. A comprehensive examination of the roles of WAKs in plant organogenesis and morphogenesis should be coupled with a deeper understanding of the mechanisms regulating vascular tissue development. Compared to the signaling pathways of WAKs during primary wall expansion, our knowledge about WAK interactions and downstream signaling pathways in SCWs is lacking. Further studies should focus on the identification of interacting proteins and downstream pathways of WAKs in secondary walls. A comparison of SCW and primary wall signaling pathways will provide a greater understanding of WAK specialization in different wall contexts. A systematic comparison of phenotypes associated with WAKs or WAKLs in future studies will aid in the identification of different functions between WAKs and WAKLs. This will facilitate knowledge of how plant cells perceive alterations in their external milieu and subsequently respond with physiological reactions. A more comprehensive understanding of WAK functions in both primary and secondary walls can also facilitate the development of novel strategies for plant growth regulation and breeding.

## Supplementary Materials

The following supporting information can be downloaded at: https://www.mdpi.com/article/10.3390/plants14030343/s1, Supplementary Figure S1. Phylogenetic tree of WAKs and WAKLs from Arabidopsis.; Supplementary Figure S2. Multiple sequence alignment comparison of WAK and WAKL proteins from Arabidopsis generated using DNAMAN.
