# Supplementary material for: Function of WAKs in Regulating Cell Wall Development and Responses to Abiotic Stress
Source: Plants (Basel). 2025 Jan 23;14(3):343. doi: 10.3390/plants14030343 (PMC11820136; doi:10.3390/plants14030343)
Supplement: Supplementary file 1 [file plants-14-00343-s001.zip › plants-3415535-supplementary.pdf]

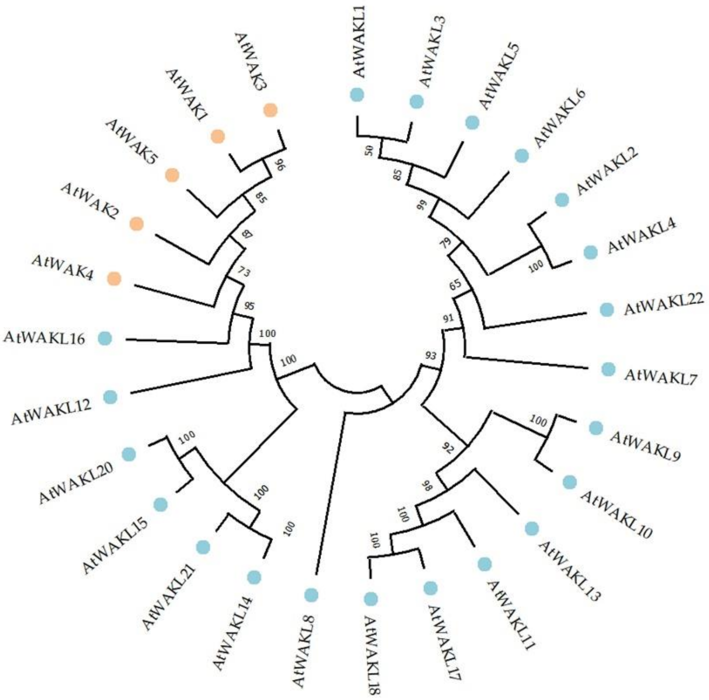

Figure S1. Phylogenetic tree of WAKs and WAKLs from Arabidopsis. The un-rooted tree was generated using MEGA11 with the neighbor-joining method (1,000 bootstrap replicates). The orange and blue dots represent WAK and WAKL proteins respectively.

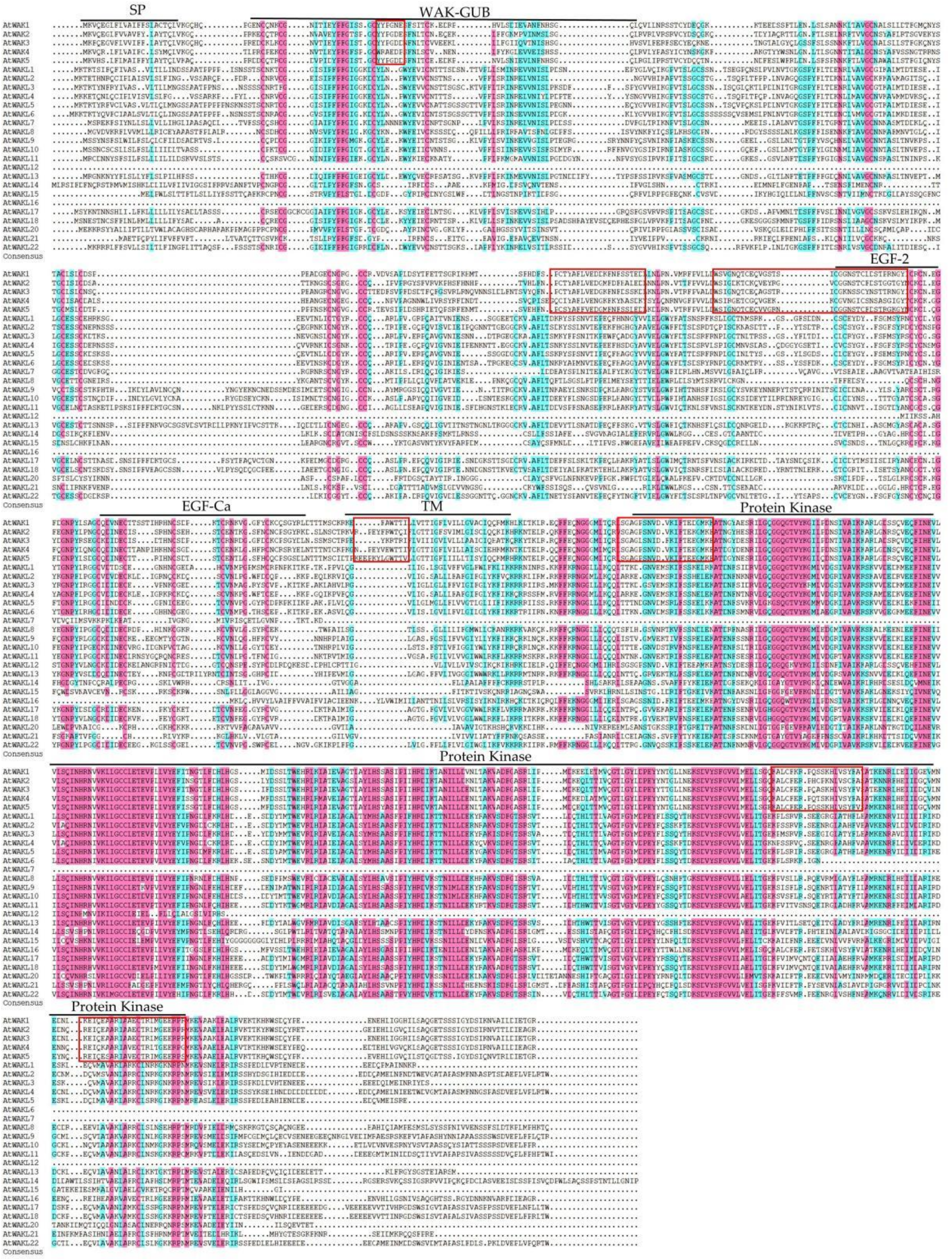

Figure S2. Multiple sequence alignment comparison of WAK and WAKL proteins from Arabidopsis generated using DNAMAN. Pink shading represents homology level greater than 75%, and blue shading greater than 50%. The signal peptide (SP), WAK-GUB domain (WAK-GUB), EGF2-like region (EGF-2), calcium-binding EGF-like domain (EGF-Ca), transmembrane region (TM) and cytoplasmic protein kinase (protein kinases) are indicated above the sequence alignment. The red boxed regions represent amino acid sequences with significant differences between AtWAKs and AtWAKLs.
